# Objective structured teaching examination (OSTE):an underused tool developed to assess clinical teaching skills. A narrative review of the literature

**DOI:** 10.1590/1516-3180.2018.0308161118

**Published:** 2019-06-10

**Authors:** Saadallah Azor Fakhouri, Maria do Patrocínio Tenório Nunes

**Affiliations:** I MD, PhD. Geriatrician, Department of Internal Medicine, Universidade Federal de Uberlândia (UFU), Uberlândia (MG), Brazil.; II MD, PhD. Associate Professor, Department of Internal Medicine, Faculdade de Medicina da Universidade de São Paulo (FMUSP), São Paulo (SP), Brazil.

**Keywords:** Educational, medical, Educational measurement, Professional competence

## Abstract

**BACKGROUND:**

There are plenty of options for evaluating medical students and medical residents’ clinical skills. Objective structured clinical evaluations (OSCEs) have emerged as a powerful and reliable tool for assessing multiple cognition domains of clinical expertise. In the same way as OSCEs have emerged to assess clinical skills, objective structured teaching evaluations (OSTEs) have come to light as promising and unbiased interventions for evaluating the act of clinical teaching.

**DESIGN AND SETTING:**

Narrative review developed at Universidade Federal de Uberlândia, Brazil.

**METHODS:**

We searched the literature regarding OSTEs using the MEDLINE (via PubMed) and LILACS (viaBiblioteca Virtual em Saude) databases. The SciELO library was also searched for Brazilian papers. Systematic reviews, reviews and randomized controlled trials specifically assessing how OSTEs performed in relation to development of academic staff and medical residents were then selected.

**RESULTS:**

Our search retrieved 178 papers, of which 40 were considered eligible for intensive review. Most of the studies selected reported positive effects from OSTE activities. However, there was little quantitative data to gauge the impact of OSTEs on improvement of teaching skills.

**CONCLUSIONS:**

Considering that OSCEs have become a widely used tool for assessing medical students’ and residents’ clinical skills, it is high time to incorporate OSTEs for evaluating teaching skills in Brazil. Encouraging data to support implementation of this assessment tool in this country is available from abroad. The net benefit from this would possibly encompass medical students, residents and academic staff, through bringing awareness about the importance of excelling in teaching skills.

## INTRODUCTION

Teaching is a complex activity for which multiple methods are needed in order to properly evaluate it. Teaching performance can be directly examined and students’ results can be highlighted. All assessment methods have their strong and weak points and any method used in isolation will provide an incomplete image of academic staff members’ teaching skills.

Goe et al.^1^ described five measurable aspects of teaching efficacy. They asked whether the teaching was doing the following: (1) helping with learning; (2) contributing towards enhancement of academic, social and attitudinal performance; (3) using multiple resources to engage students in learning opportunities; (4) promoting civic values and respect for diversity; and (5)collaborating with the institution, peers and families to help students succeed, particularly those with a higher chance of failing.

The method most used for assessing the act of teaching comprises direct observation of the activity. This can be done by directors, other academic staff members (supervisors) or external evaluators (locally or with the aid of video-recorded performances). These evaluations are, in general, more time and resource-consuming than are estimates centered on tests that are based on teaching efficacy, such as national and international examinations on students’ performance. Standardized tests provide a more economical and efficient way of making reliable measurements of the content acquired by students.^2^

Evaluations are routine events in the lives of university students worldwide. Their methods take different shapes, but one of the methods within medical education consists of assessments of simulated situations that have been developed specifically to assess clinical skills. Since the famous neurologist Howard Barrows^3^ used trained actors to evaluate his residents’ examination skills in a simulated environment in the 1960s, use of this assessment method has spread fast in the academic community.

Nowadays, medical schools are not alone in using simulated events. Training courses for a wide diversity of careers, especially health-related courses, have embraced this resource as a means of evaluation.

The authors of the present narrative review are working on the development of residents as teachers, using OSTE as a means of evaluation. Considering the need for refinement in academic staff development and the importance of medical residents’ teaching skills awareness, we judged it to be of utmost interest to introduce, report on and disseminate the concept of assessment of clinical teaching skills. In this paper, we describe the main evidence that provides support for implementing the OSTE method as an objective, summative and formative tool.

## OBJECTIVE

The main objectives of this narrative review were to introduce the concept of evaluating the act of teaching, through systematic observation of a simulated activity; and to summarize the evidence that provides support for dissemination of the “objective structured teaching examination/evaluation/exercise/encounter” method (OSTE).

## METHODS

We conducted a review of the literature using the MEDLINE database (via PubMed) and the LILACS database (via Biblioteca Virtual em Saúde) to extract relevant articles that describe simulated activities that were performed with the specific aim of evaluating teaching skills. We also searched the SciELO library for Brazilian papers. The key words used in the literature search included “objective structured teaching examination”, “objective structured teaching evaluation”, “objective structured teaching encounter”, “objective structured teaching exam”, “observed structured teaching examination” and teaching AND evaluation. The MeSH terms used in the search were educational measurement/methods, professional competence, teaching/methods and teaching/standards.

The relevant publications included original articles, systematic reviews, critical reviews, randomized controlled trials, guidelines from medical education experts and material from pioneering authors in this field. Attention was given to papers focusing on pre and post-OSTE intervention designs, validity and reliability assessments, experimental work and publications describing the process of creating OSTE scenarios.

## RESULTS

Of all identified papers, 40contained information regarding the specific topic of evaluating clinical teaching skills through objective and structured simulated activities. Searches for primary publications referenced in other articles were also included. These 40 articles with relevant information were selected for intensive review and were analyzed by two authors (SAF and MPTN). The search in LILACS and SciELO using Portuguese key words did not yield any results regarding the specific topic of interest. Table 1 describes the number of texts extracted from each database.

**Table 1*.** Evolution of objective structured teaching examinations (OSTEs) over time

Year	Author	Name attributed to activity	Target audience
1992	Simpson et al.^4^	SATS: standardized ambulatory teaching situations	Academic staff member
1994	Orlander et al.^5^	CFE: clinical feedback exercise	Residents
1994	Lesky et al.^6^	OSTE: objective structured teaching exercise	Academic staff member
1997	Kachur et al.^9^	Multiple-station exam/teaching exercise	Academic staff member
1998	Dunnington et al.^10^	OSTE: objective structured teaching evaluation	Residents
1998	Prislin et al.^7^	OSTE: objective structured teaching evaluation	Academic staff member
2001	Schol et al.^11^	MSTAT: multiple-station teaching assessment test	Academic staff member

*Inspired by the timeline developed by Elizabeth Kachur.

The majority of the studies included in this review reported that the participants (academic staff members and residents alike) gave positive responses regarding the activities involving OSTEs. There was a trend towards lack of robust quantitative data on these activities. Nonetheless, overall, these studies recommended dissemination of OSTEs as a means of assessing clinical teaching skills. Table 2 describes the most relevant studies included in this review and summarizes their main objectives and results.

**Table 2.** Literature search in medical databases, search strategies used for each database and number of articles extracted

Database	Search strategies	Papers found
MEDLINE (via PubMed)	#1- (“objective structured teaching evaluation”[Title/Abstract]) OR (“objective structured teaching examination”[Title/Abstract]) OR (“objective structured teaching encounter”[Title/Abstract]) OR (“objective structured teaching exam”[Title/Abstract]) OR (“observed structured teaching examination”[Title/Abstract])	15
	#2-(“educational measurement/methods”[Mesh Terms]) OR (“professional competence”[MeSH Terms]) OR (“teaching”[MeSH Terms]) OR (“teaching/standards”[Mesh Terms])	175,601
	#3- #1 AND #2	13
LILACS (via Biblioteca Virtual em Saúde)	#1- (tw:(internato e residência)) AND (tw:(capacitação em serviço)) AND (tw:(treinamento por simulação)) AND (instance:”regional”) #2- (tw:(modelos educacionais)) AND (tw:(avaliação educacional)) AND (tw:(treinamento por simulaçao)) AND (instance:”regional”)	0

## DISCUSSION

### Defining the instrument

In medical education, simulated assessments of clinical skills, known as “objective structured clinical evaluations” (OSCEs) are routinely conducted. At our institutions, OSCEs are used with summative, formative and selective purposes, such as in entry contests for medical residency. In the same way in which clinical skills can be placed under scrutiny through simulated experiences, some authors have hypothesized that teaching skills could also be assessed using the same principles.^4^^,^^5^^,^^6^^,^^7^

Clinical training is vital for producing medical staff who are capable of delivering high quality assistance. Successful clinical teachers actively engage their students in patient care and provide constructive counseling and feedback.

Objective structured teaching examinations/encounters/evaluations/exercises (OSTEs) have emerged as promising and unbiased interventions for assessing the act of teaching. The reasons that are commonly given for using OSTEs as a means of assessing the teaching skills of teachers and facilitators are that this method promotes rapid and rigorous assessment and that it enables practicing of specific skills in a realistic atmosphere with immediate feedback and potential benefits for raters.^8^

The first studies using OSTEs were started during the 1990s. Table 3 details how simulated activities designed to assess teaching skills have evolved over the years. ^4^^,^^5^^,^^6^^,^^7^^,^^9^^,^^10^^,^^11^^,^^12^^,^^13^^,^^14^^,^^15^ This table presents a modified version of material that was produced in a workshop conducted by Wamsley et al.,^8^ at the University of California, in 2006. These authors specifically described OSTEs as a novel tool that was designed to enhance teaching skills and techniques.

**Table 3.** Studies of relevance to the objective structured teaching examination (OSTE) method, included in the present review

Authors	Year	Study type	Participants (n)	Specialty	Objectives	Conclusions
Orlander et al.^5^	1994	Qualitative/ experimental	23 residents and 47 interns	Internal medicine	To obtain residents’ and interns’ opinions after participating in a clinical feedback exercise	Residents reported better attitudes towards clinical teaching skills and contact with interns
Lesky et al.^6^	1994	Qualitative/ experimental	Ambulatorial preceptors and standardized students (sample not informed)	Not informed	To develop an ambulatorial preceptorship program and help preceptors to adopt a student-centered teaching approach	Preceptors who participated in the activity reported better comprehension of their clinical teaching competencies
Schol et al.^11^	2001	Validity and reliability	35 residents acting as preceptors	General practice	To evaluate the validity and reliability of an evaluation with seven OSTE scenarios	This type of assessment provides important information about the levels of competency in clinical teaching
Morrison et. al^13^	2002	Validity and reliability	23 second-year residents	Internal medicine, pediatrics and family medicine	To evaluate the validity and reliability of an eight-station OSTE that was developed specifically for residents involved in teaching activities	Cronbach’s alpha > 0.9 for all eight stations; 92% of the residents reported that the stations realistically reflected important clinical teaching skills
Stone et al.^14^	2003	Pre and post-intervention design	40 teachers	Family medicine, pediatrics and family medicine	To develop and implement an OSTE to assess an academic staff development module	OSTEs can be more sensitive for detecting skills that are more readily applicable to daily practice
Sturpe et al.^17^	2014	Descriptive	NA	NA	To describe how to develop a scenario to evaluate clinical skills	When used together with other teaching strategies, OSTEs can enhance academic staff members’ teaching strategies
Cerrone et al.^21^	2017	Non-randomized intervention	80 chief-residents	15 specialties	To develop a program capable of enhancing chief-residents’ emotional intelligence	There was a statistically significant improvement in OSTE scores after the intervention
Zackoff et al.^23^	2015	Prospective, with intervention group and blinded raters	49 residents	Pediatrics	To create an OSTE capable of detecting changes in residents’ teaching skills after participation in a teaching skill development program	The OSTE showed efficacy in detecting changes in clinical teaching skills
Steinert et al.^24^	2006	Systematic review	NA	Multiple	To describe the evidence that gives support to programs aimed towards developing teaching skills	Participants generally reported positive changes after specific interventions, but methodological limitations remain and need to be addressed
McAndrew et al.^25^	2011	Descriptive	12 teachers	Dentistry	To describe the creation of an OSTE to assess an academic staff development program within dentistry	Use of OSTEs not only enhances the levels of academic staff development programs, but also enriches efforts to foster teaching within dentistry
McCutcheon et al.^26^	2017	Pilot, qualitative, pre and post-OSTE	23 preceptors	Pharmacy	To evaluate interprofessional teaching skills	Pharmacy preceptors demonstrated better teaching skills and self-confidence after participating in OSTEs
Wamsley et al.^31^	2005	Qualitative/ experimental	16 teachers	Internal medicine, pediatrics and family medicine	To develop teaching skills among academic staff members in different specialties	Teachers reported that they would change their teaching styles after participating in OSTEs; standardized students reported more interest in teaching after the activity
Trowbridge et al.^33^	2011	Systematic review	NA	Multiple	To describe the use of OSTEs and the evidence that gives support for their use	OSTEs are a promising innovation with potential application in assessment and promotion of clinical teaching; new research is necessary to define the designs of appropriate stations

NA = not applicable.

OSTEs consist of simulated scenarios of teaching environments with students and have the aim of providing immediate objective feedback to the teacher or medical resident participating in the activity.^16^ Most of the literature has described OSTEs as 10 to 15-minute encounters in which the students interact with a teacher in video-recorded sessions that are then discussed in large debriefing groups.^17^ They provide an opportunity for teachers to show how they teach and interact with standardized students in a controlled and thus protected scenario. There is the possibility of enacting daily situations and repeating them as necessary.

During an OSTE activity, the participants go through sequential stations, each consisting of a different clinical teaching situation. Standardized students are used (trained to present a prototypical teaching challenge consistently across many meetings with different teachers), and a simulated patient can be included, depending on the purpose of the specific station. Both verbal and non-verbal communication skills are evaluated. Feedback is given about different aspects of the teacher’s performance. Feedback from the standardized student can also be given. The materials needed for performing the activity include descriptions of the situations in every detail, good training and precise orientations for the standardized student and instructors, and a reliable and validated instrument for rating the OSTE. Standardization is expected to occur because the real students or trained actors follow a script with predefined objectives.

### Instrument applications

Teaching evaluations in OSTE scenarios are used with distinct purposes in American academic institutions. Some authors have used OSTEs to evaluate the teaching skills of medical preceptors, academic staff members at multiple schools and residents.^7^^,^^10^^,^^11^^,^^18^^,^^19^

McSparron et al.^20^ developed an OSTE with the specific aim of assessing academic staff members’ skills in orienting a procedure. The task consisted of guiding a standardized student who was required to place a central line in a model that had been developed for this purpose. These authors named their activity “prOSTE” and they reported that the performance of standardized students was better when the instructors offered positive feedback and suggestions for improvement and when the procedure was explained step by step. Another use for this method was to observe medical teachers or residents’ teaching skills and try to enhance them through effective feedback.

Cerrone et al.^21^ described an activity in which the aim was to use OSTEs as a means of evaluating and promoting chief residents as emotionally intelligent leaders. These authors cited emotional intelligence as a core competence of leadership that was required from these residents. The OSTE scenarios for this activity addressed professionalism, confidentiality, teamwork and escalation issues (resolution of conflicts with team members who were at a higher hierarchical level). The chief residents were divided into pairs and participated in simulations of authentic incidents that were not identified. These authors concluded by affirming that OSTEs combined with post-activity debriefing sessions provided a powerful platform through which individuals undergoing evaluation (teachers or residents) would be able to demonstrate competencies in areas that were frequently ignored, omitted or even considered “hidden”. Lastly, the strategy was also used to evaluate interventions that were designed to enhance the teaching skills of academic staff members and medical residents at different institutions.^14^^,^^22^^,^^23^

Steinert et al.^24^ reviewed 303 articles on academic staff member development programs. They specifically emphasized the importance of using OSTEs to measure the scores before and after the interventions.

### Using the instrument beyond medicine

OSTEs are nowadays not restricted to medicine or to preceptors and residents working in large university hospitals. McAndrews et al.^25^ developed an activity based on OSTEs to measure the response of academic staff members in the dentistry department of New York University, to a development program for teaching skills. Sixteen academic staff members participated in a study measuring scores before and after the intervention. Three stations were created to evaluate 15domains of teaching. There was a statistically significant improvement in the scores from all the domains tested, after exposure to an intervention based on academic staff member development.

McCutcheon et al.^26^ described a study in which the aim was to training pharmacy preceptors in interprofessional educational activities. One OSTE station was created in such a way that two standardized students (one from nursing and one from pharmacy) interacted with a patient who had been diagnosed with asthma. Analysis on the data obtained showed that there were statistically significant improvements in all the 15 self-confidence items for which the participants gave responses. These authors highlighted the following items as resulting in significant improvements in students’ abilities: guiding students from different specialties, facilitating a simulated activity, leading a debriefing session and discussing the core competencies in interprofessional education.

### Rating OSTE participants

OSTEs provide highly versatile evaluations. They can be used for either educational or assessment purposes. Currently, OSTEs form the gold-standard instrument for assessing medical residents’ teaching skills.^25^ They have also become the most accurate measurement method for evaluating academic staff member development programs.^24^

To provide unbiased, valid and reliable scores, activities such as OSTEs require appropriate measurement instruments. It is imperative that these instruments should have highly accurate measurement properties, so that they can contribute reliable and relevant feedback regarding the qualities and deficiencies demonstrated by the individuals who are undergoing evaluation.

Review studies have identified more than 32 different instruments that were developed to score simulated activities within clinical teaching. These instruments are an essential part of the process of continuous qualification in medical education and development of clinical teaching competencies.^27^

Two scoring instruments have been validated and are widely used by the academic community: (1) the “Stanford Faculty Development Program” (SFDP26);^28^ and (2) the “System for Evaluation of Teaching Qualities” (SETQ).^29^ The SFDP26 was originally developed in the United States and evaluates the following teaching skills: (1) establishment of the learning environment; (2) session control; (3) communication of learning goals; (4) facilitation of retention and understanding; (5) evaluation of previous knowledge, (6) promotion of self-directed learning; and (7) giving feedback.

### Instrument advantages:

Irby et al.^30^ described the main reasons for using OSTEs: (1) rapid and rigorous evaluation of clinical teaching skills; (2) provision of time for practicing of specific teaching skills; (3) creation of a practical and realistic atmosphere; (4) provision of immediate feedback; and (5) potential for benefiting the standardized students, who acquire additional knowledge while they rehearse for the stations and while they give feedback to the person undergoing evaluation.

According to Wamsley et al.,^31^ teaching skills are deemed to be of good quality, in most scenarios, through students’ personal opinions. This type of evaluation is susceptible to the teacher’s charisma and to her/his communication skills. In this circumstance, the picture of what constitutes a good teacher can be contaminated by personal interactions in daily activities, either in a classroom or in hospital wards. This interaction bringing a positive or negative bias. OSTEs have the advantage of attenuating this potential contamination through specifically assessing the teacher’s professional performance. This can be achieved using standardized situations, instruments, students and even patients.

Another attractive feature of OSTEs is that they provide the opportunity for an immediate debriefing session after the activity. The debriefing process is considered by many researchers to be the most important component of any learning experience based on simulation. It relates directly to the learner’s capacity to reflect on his/her performance in the activity.

Decker et al.^32^ stated that the learning process is dependent on the interaction between experience and reflection. Regarding the reflection process, these authors took the view that this comprised conscious consideration of the meanings and implications that arise from an action. All these processes would lead to assimilation of knowledge, skills and attitudes that did not exist before the intervention. These authors concluded by citing the necessary conditions for an effective debriefing session: (1) facilitated by a competent individual; (2) conducted in an environment that favors knowledge acquisition and that guarantees confidentiality, trust, open communication, self-analysis and reflection; (3) facilitated by someone who directly observed the simulation experience; (4) based on a work plan specifically structured for that task; and (5) congruent with the participants’ objectives and the previously established results.

### Instrument limitations

According to the literature reviewed here, the main limitations on developing OSTEs are the scarcity of financial and human resources for implementing the activities and the lack of time that can be attributed to tasks that are deemed “less important”. Training standardized students or hiring professional actors for the activities can be a limiting factor in institutions in which simulations are not part of the local culture. There are also no data on the financial impact of this kind of activity or on its cost effectiveness.

A systematic review on the use and efficacy of OSTEs, published in 2011, did not find much evidence in the form of quantitative data to gauge the impact that this method might have in relation to improvement of teaching skills.^33^ Nonetheless, most participants reported that they benefited from the activities. Thesame authors also cited the lack of uniformity in the scripts for the scenarios that were used. The reliability of the rating instruments was moderate to high in most of the studies and the rating scores appeared not to be related to rater background, i.e. academic staff member or student. Only four studies specifically evaluated station validity and, thus, little evidence regarding OSTE validity up to the date of that review was available.^11^^,^^13^^,^^18^^,^^34^

### Brazilian studies

Our search using Portuguese key words in the LILACS and SciELO databases did not yield any results. To our knowledge, there has been no experience of using the OSTE method in Brazil. The present review demonstrates that there is little or no knowledge about how to apply the OSTE method in Brazil, despite all the positive remarks regarding this activity. We have now managed to successfully develop OSTE scenarios within a doctoral project that is still in progress. Four scenarios were created, in which discussion of cases in emergency, ambulatory and surgical ward settings was simulated and a simple procedure was taught. We translated the SFDP26 instrument into Portuguese and then validated it using a specialists’ panel; we trained arts students as standardized medicine pupils; and finally, we received voluntary help from medical residents who performed in the proposed simulations (data not yet published).

This narrative review forms part of a doctoral project that is still in progress. Its aim is to assess residents’ clinical teaching competencies before and after an intervention based on the one-minute preceptor technique.^35^ The OSTE method was presented, based on our previously cited experience, at the Brazilian Congress of Medical Education in 2017, as a workshop.

Adequate assessment of teachers’ performance gives them the opportunity to reflect on their teaching skills and consequently evolve during the process of knowledge acquisition. Teachers desire and need feedback, not only in relation to the time that they spend in the classroom, but also in relation to how this enhances the educational results. Evaluation systems directed towards teachers are frequently proposed, in order to provide feedback and guide the evolution of professional practice.^36^

Assessment of academic staff members, preceptors and medical residents is a subject of major controversy not only in Brazil. Webelieve that most teachers with well-consolidated careers would feel uneasy about having their skills put under scrutiny from their peers or students. This may explain why the word evaluation, contained in the acronym OSTE, is frequently changed to encounter, exercise or situation.

In the same way in which OSCEs have been brought into Brazilian settings as a tool for assessing medical students and residents, it is also proving possible to develop the dynamics of OSTEs within our practice. Some useful papers have dealt specifically with the process of creating OSTE stations, and these form an excellent starting point. Boillat et al.^16^ presented 12 tips for constructing activities based on simulation, for assessing clinical teaching skills (Table 4). There are potential benefits from OSTEs at all educational levels, given that evaluation of reactions (the lowest level on the Kirkpatrick scale^37^) is being superseded, such that the level of understanding of attitudes and behavioral change is now being reached.

**Table 4*.** Tips for producing an objective structured teaching examination (OSTE) scenario

1	Establish specific goals for the activity in advance
2	Determine context and target audience for the activity
3	Identify specific teaching skills to be evaluated
4	Set the scenario: use real cases based on situations that occurred in the place where the activity is being performed
5	Develop the assessment tool: checklists, self-evaluations and those to be performed by standardized students or peers
6	Choose the standardized student: medicine or other pupils, trained actors, academic staff members or other institutional staff
7	Train the standardized student according to the scenario and teaching skill to be assessed
8	Test the scenario before the main event to correct potential errors and maximize case fidelity
9	Protect the teacher undergoing evaluation: strictly follow the principles of effective debriefing and feedback to reduce stress from the activity
10	Incorporate this simulation method into academic staff development programs in the specific context of your institution
11	Emphasize the positive aspects of the method to enhance adherence by academic staff members or residents
12	Evaluate the activity after concluding the simulations: use pre and post-activity interviews, written evaluations or qualitative comments

*Modified from Boillat et al.^16^

## CONCLUSIONS

Teaching is a complex activity that requires multiple methods for complete assessment. In the present review, we elected to examine a standardized observational method: OSTEs. Considering that OSCEs have become a widely used tool for assessing medical students’ and residents’ clinical skills, it is high time to incorporate OSTEs for evaluating teaching skills in Brazil. Encouraging data to support implementation of this assessment tool in this country is available from abroad. The net benefit from this would possibly encompass medical students, residents and academic staff, through bringing awareness about the importance of excelling in teaching skills.
